# Comparing Foundation Doctors’ Confidence and Dermatologists’ Expectations in Inpatient Referrals: A Multi-site Survey

**DOI:** 10.7759/cureus.93830

**Published:** 2025-10-04

**Authors:** Kimia S Khorshid, Arash Fattahi, Mohsen Khorshid

**Affiliations:** 1 Dermatology, Basildon University Hospital NHS Trust, Basildon, GBR; 2 Dermatology, Northampton General Hospital NHS Trust, Northampton, GBR; 3 Dermatology, Mid and South Essex NHS Foundation Trust, Basildon, GBR

**Keywords:** dermatology training, emollients, foundation doctors, inpatient referrals, medical education in dermatology, perceived confidence, referral quality, united kingdom (uk)

## Abstract

Background: Inpatient dermatology referrals rely on non-dermatologist doctors to recognise and communicate skin problems effectively. Limited dermatology training during medical school may leave newly qualified doctors underprepared for this responsibility.

Objectives: This study aimed to evaluate foundation doctors' confidence in core dermatological skills and knowledge and to compare these with dermatologists' expectations based on observed inpatient referral quality across two UK hospital sites.

Methods: A cross-sectional survey was conducted at Basildon University Hospital and Northampton General Hospital in England. Foundation year doctors (n = 87) completed questionnaires assessing confidence (on Likert scales 1-5) in describing skin lesions, identifying urgent dermatological conditions, knowing what information to include in referrals, and using common treatments. Dermatologists (n = 8) were surveyed on expected referral information, common deficiencies, and examples of inappropriate referrals. Descriptive statistics were used to compare responses.

Results: Across both sites, most foundation doctors reported low confidence in describing skin lesions and identifying serious dermatoses: only 8-29% rated themselves "confident" or "extremely confident." Over 65% were not confident in using different emollients (only 2-10% felt confident). Only ~30% of foundation doctors were aware of the British Association of Dermatologists (BAD) handbook. Dermatologists consistently expected detailed clinical information in referrals but noted that many lacked these elements. Common conditions like eczema, psoriasis, scabies, and drug eruptions were cited as being referred inappropriately.

Conclusions: Foundation doctors reported limited confidence in fundamental dermatological assessment and management skills, reflecting inadequate undergraduate dermatology training. Dermatologists observed corresponding deficiencies in referral quality. This mismatch may lead to suboptimal patient management and unnecessary specialist referrals. Improving dermatology education for medical students and resident doctors is recommended, alongside clearer referral guidelines and continued dermatologist feedback.

## Introduction

Dermatological conditions are frequently encountered in hospital inpatients, yet many resident doctors feel ill-equipped to assess and manage them. UK undergraduate curricula give limited emphasis to dermatology. A recent scoping review of curricula in the UK, USA, Canada, and Australia shows the pooled average time for undergraduate dermatology has fallen to 19.2 hours today versus 33.8 hours 40 years ago [1]. As a result, newly qualified doctors often lack confidence in fundamental skills such as describing rashes, proposing a differential diagnosis, and initiating basic treatment.

Arguably, this variation in teaching time results in inconsistencies in what is taught. Davies and Burge audited UK undergraduate curricula against British Association of Dermatologists (BAD) recommendations and found significant omissions in basic learning outcomes at some medical schools [2]. For example, some schools failed to include examination of the skin or teaching common conditions such as acne in their curriculum, and ultimately, such an inconsistency in teaching affects clinical competence. A more recent audit by Yaakub et al. similarly found persistent gaps in dermatology teaching across UK medical schools, with variations in clinical exposure and assessment methods [3].

This is supported by Hussain et al., who found that there was a clear lack of confidence displayed by resident doctors in basic skills such as describing a rash, with only eight percent feeling confident in initiating basic treatment for a skin presentation [4]. Similarly, a study focusing on foundation year one (FYone) doctors found that many did not feel sufficiently prepared to manage skin disease, correlating with limited undergraduate exposure [5]. This educational gap may result in missed serious skin conditions or delayed management by non-dermatologist doctors [6]. The situation has been shown to persist despite curriculum reforms, as documented by Burge's comprehensive survey of UK medical schools, which revealed that dermatology teaching time remained static over decades despite increasing skin disease burden [7].

In the inpatient setting, significant knowledge gaps in dermatology among non-specialist doctors are reflected in the increasing demand for specialist consultations [6]. Prior research has highlighted common shortcomings in inpatient dermatology referrals. Cnudde et al. audited 14 years of inpatient referrals in Wales and found that referring teams failed to offer any differential diagnosis in 41% of cases, and only 29% of referrals had a correct initial diagnosis [6]. Notably, even very common conditions such as eczema were often underdiagnosed or mismanaged by admitting teams. In over half of referrals (54%), the primary team had not mentioned starting any treatment for the skin issue [6]. These findings suggest a lack of confidence and knowledge among referring doctors, which aligns with anecdotal reports from dermatologists that key information is frequently missing from referrals and that many of these could be avoided or improved with better training.

Improving the interface between frontline resident doctors and dermatology services is important for patient safety and service efficiency. Inadequate referral information or unnecessary referrals can delay diagnosis, lead to inappropriate treatments, and overburden specialist services [8]. Recent efforts to address these issues include the development of educational tools and algorithms to improve referral quality, as demonstrated by Middleton and Dolan's work on descriptive terminology training [9].

Within the UK, the Foundation Programme offers an opportunity to reinforce dermatology training for new doctors. Yet dermatology is not a mandatory rotation, and many FYone/FYtwo doctors may have had minimal exposure beyond medical school. Some hospitals offer teaching or handbooks (e.g., the free BAD Handbook), but the uptake and awareness of these resources are variable. The implementation of specialty-specific curricula faces numerous barriers, as identified by Sharma et al., including competing service commitments and a lack of recognition for educational leadership [10].

To address this gap, we conducted a multi-site survey at two district general hospitals to evaluate the confidence of FYone-FYtwo doctors in key dermatological tasks relevant to inpatient care, and to contrast these self-assessments with dermatologists' perspectives on referral quality. By identifying specific disparities, for example, in describing skin lesions or recognising urgent conditions, we aim to inform targeted educational interventions for undergraduates and postgraduates.

## Materials and methods

Study design and setting

We carried out a descriptive cross-sectional survey study at two National Health Service (NHS) hospitals in England: Basildon University Hospital in Essex and Northampton General Hospital (NGH) in Northamptonshire. These sites were chosen to provide independent samples for a collaborative multi-site perspective. Both hospitals are district general hospitals with dedicated dermatology departments receiving inpatient referrals from various specialties. Participation was voluntary and anonymous.

Participants

Two groups were targeted: FY doctors (FYone and FYtwo) working in each hospital and dermatologists (registrars and consultants) at each site. Inclusion criteria for resident doctors were being in a Foundation Programme post at the study sites during the survey period; there were no exclusion criteria. All dermatology specialists at Basildon and NGH were invited to participate. In total, 52 foundation doctors responded at Basildon and 35 at NGH. Eight specialist responses were received (five from Basildon and three from NGH).

Survey instruments

We designed parallel questionnaires for foundation doctors and for dermatologists, with complementary topics. The foundation doctor survey consisted of five main questions focusing on areas of dermatological knowledge or skills relevant to inpatient referrals, four of which were rated on a Likert scale [11] from one "Not confident at all" to five "Extremely confident", and one was a simple yes/no question. The dermatologist's survey asked three open-ended questions. Details of the questions are outlined in the results section.

Data collection

Data collection took place in December 2024. Author-made surveys were distributed within foundation doctor teaching sessions via QR codes, which opened Google Forms (Google LLC, Mountain View, California, USA). The questionnaires were anonymous to encourage honest self-assessment. Dermatologists were emailed their author-made surveys, and they provided qualitative feedback, which was analysed without attribution to any individual. All responses were collected within a four-week period, at which point the surveys were closed. There was no identifying patient data involved; the study was categorised as a service evaluation/educational needs assessment and did not require formal ethical committee review under local guidelines.

Data analysis

Quantitative data from the foundation doctor surveys were entered into Microsoft Excel 365 (Microsoft Corp., Washington, USA) and analysed descriptively. For each Likert-scale question, we calculated the distribution of responses (count and percentage of respondents choosing each option 1-5) at each site. Given the sample sizes, formal statistical comparison between sites was limited to descriptive comparisons.

Qualitative responses from dermatologists were compiled and analysed using thematic analysis. Initial codes were developed through repeated reading of the data, with recurring ideas identified and labelled. These codes were then reviewed and grouped into broader themes through discussion among the research team to ensure consistency. Representative quotes were extracted to illustrate key points. Finally, the qualitative themes were then compared to the residents' self-reported confidence domains to identify alignment or disparities.

## Results

A total of 87 foundation doctors (FYone/FYtwo) across the two sites completed the survey (52 from Basildon, 35 from Northampton). Table 1 summarises their responses to the confidence questions. Overall, there was a clear trend of low self-assessed confidence in dermatology-related skills. Table 2 summarises dermatologists' expectations for inpatient referrals and highlights the common deficiencies observed in actual referral practice. Furthermore, Appendix A lists the full foundation doctor questionnaire, with Appendices B-K in the appendices displaying their full responses. Appendix L in the appendices lists the full dermatologist questionnaire, with Appendices M-N in the appendices displaying their full responses.

**Table 1:** Foundation doctor survey responses Foundation year doctors' self-reported confidence in dermatology skills (Basildon vs. Northampton). Values are number of respondents (% of group). Likert scale [11]: one = not confident at all, five = extremely confident.

Question (abridged)	Rating	Basildon (n = 52)	Northampton (n = 35)
1. Confidence in describing abnormal skin lesions	1 – Not confident at all	7 (13%)	6 (17%)
	2	20 (39%)	8 (23%)
	3	21 (40%)	18 (51%)
	4	3 (6%)	2 (6%)
	5 – Extremely confident	1 (2%)	1 (3%)
2. Confidence in identifying red-flag skin presentations	1 – Not confident at all	4 (8%)	4 (11%)
	2	13 (25%)	9 (26%)
	3	20 (39%)	16 (46%)
	4	13 (25%)	5 (14%)
	5 – Extremely confident	2 (4%)	1 (3%)
3. Confident in knowing what info to include in referral	1 – Not confident at all	5 (10%)	3 (9%)
	2	15 (29%)	13 (37%)
	3	19 (37%)	11 (31%)
	4	10 (19%)	6 (17%)
	5 – Extremely confident	2 (4%)	2 (6%)
4. Confidence in using different forms of emollients	1 – Not confident at all	16 (31%)	8 (23%)
	2	17 (33%)	16 (46%)
	3	13 (25%)	10 (29%)
	4	4 (8%)	1 (3%)
	5 – Extremely confident	1 (2%)	0 (0%)
5. Aware of BAD handbook (Yes/No)	Yes	15 (29%)	11 (31%)
	No	37 (71%)	24 (69%)

**Table 2:** Dermatologists’ expectations for inpatient referrals vs. common deficiencies observed

Dermatologists' expectations (Referral should include...)	Common deficiencies noted in actual referrals
Detailed lesion description - morphology, size, colour, etc., using dermatologic descriptors if possible.	Descriptions often vague: e.g., "rash on arm" without characterisation. Important details like extent (percentage of body surface) or distribution, specific lesion type are frequently missing.
Relevant history of skin complaint - Duration, progression, symptoms (itch/pain), precipitating factors, triggers, similar past rashes.	History is incomplete: Many referrals omit the timeline or symptoms. Sometimes no mention of how long the rash has been present or of symptoms, making triage difficult.
Patient background - Key medical history, current medications, allergies, and any recent changes (new drug started, etc.).	Missing context: Referrals occasionally lack mention of relevant meds (e.g., "patient on chemotherapy" or "started antibiotic X one week ago") that could be crucial to diagnosis. Allergy status is sometimes not stated.
Initial management steps - Treatments or interventions already done (topical or systemic) and response, if any.	No info on treatments tried: Dermatologists often see that emollients or topical steroids weren't started, or if they were, the referral doesn't mention it. Thus, specialists cannot tell if a basic measure has been attempted or not.
Working diagnosis or differential - Referring team's suspected diagnosis or main concern (even if tentative).	Lack of differential: Many referrals have no proposed diagnosis. Dermatologists note that in the majority of cases, residents avoid committing to a diagnosis, which could reflect a lack of confidence. Even an incorrect guess can provide insight into what the referrer is thinking; its absence is a lost opportunity for communication.
Urgency and red flags - An indication of severity (e.g., vital sign abnormalities, rapidly worsening rash, mucosal involvement).	Underestimation of severity: Dermatologists recalled instances where referrals did not convey how sick the patient was. For example, a note might not mention that the patient was febrile or that >90% of skin was affected (erythroderma), which are critical details. Resident doctors may not recognise the gravity and thus fail to flag urgency in the referral.

Foundation doctor survey responses

Few foundation doctors considered themselves highly confident. For describing skin lesions, only one out of 52 Basildon respondents (2%) and one out of 35 at NGH (3%) felt "extremely confident." The majority gave themselves a two or three out of five. In fact, combining the lowest two categories (score 1 or 2) as an indicator of low confidence, 52% of Basildon residents and 40% of Northampton residents reported low confidence in describing skin lesions.

Red-flag identification showed a similar trend. At Basildon, about one-third rated low confidence (1-2) while 29% rated themselves confident (4-5). At Northampton, 37% were low and only 17% high in confidence. In other words, at both hospitals, fewer than one in three resident doctors felt confident in recognising potentially serious skin conditions.

For knowledge of what information to include in a referral, the distribution again skewed toward lower confidence. Approximately 39% (Basildon) and 46% (NGH) were not confident (rating 1-2) that they knew what details to provide when referring a patient to dermatology. Only around 23% felt confident (rating 4-5) on this aspect at either site.

Question four revealed the lowest confidence levels: understanding and using different types of emollients. At Basildon, 64% admitted low confidence (ratings 1-2) in their knowledge of emollients, and at NGH, this was 69% (with notably zero respondents feeling "extremely confident" at NGH). Only five individuals (10%) at Basildon and just one person (three percent) at NGH felt confident (score 4 or 5) about emollient use.

Finally, awareness of the BAD handbook was low at both sites. Around 29% of Basildon and 31% of NGH respondents answered "Yes" (they were aware of this resource), whereas roughly 70% were not aware (Table 1).

Dermatologists' perspectives

Dermatologists from the two sites provided qualitative insights that complement the resident doctors' self-assessments. Their responses underscored a significant disparity between the information they expect in referrals and what is often provided by referring doctors (Table 2).

When asked "What information would you like to have when you review an inpatient referral?", the specialists emphasised the need for a thorough background and description. Common points included: The patient's medical history and drug history, the reason for the current admission, a clear description of the skin problem (morphology, distribution, onset, and associated symptoms), any treatments already tried, and an assessment of urgency. One specialist noted they expect differential diagnoses to be suggested, and even having photographs of the rash can be useful.

In response to "What information do you commonly find lacking within inpatient referrals?", the comments directly mirror the areas of resident doctors' low confidence. Frequently missing elements were previous medical history and medication details, details of the presenting complaint, and above all, an accurate skin description. One dermatologist lamented that "Everything is a rash!!" - highlighting frustration that referrals often label the problem generically without characterisation (e.g., not specifying if a rash is urticarial, vesicular, purpuric, etc.).

The third question to the dermatologists asked for "recurring inappropriate inpatient referrals which could be resolved with better education", and to specify examples. Several specialists identified common dermatological conditions that, in their view, should not routinely require specialist referral if the parent team had more dermatology training. Examples given were: Drug eruptions (drug rashes), where one dermatologist felt these are often referred "for no particular reason"; stable psoriasis or eczema flares; scabies; and leg ulcers. These were cited as conditions frequently referred to dermatology despite being generalist-manageable (Table 2).

Comparison of sites (Basildon vs. Northampton)

The multi-site aspect of the study allows us to see if these trends were site-specific or widespread. The quantitative results in Table 1 show that both Basildon and Northampton foundation doctors have remarkably similar confidence profiles, with only minor differences. For example, Basildon residents had slightly higher confidence in red-flag identification than their Northampton counterparts (29% vs. 17% reporting scores 4-5). These differences may reflect individual or cohort variability; there was no consistent pattern of one site being more confident across all domains.

Specialist feedback from the two sites was also comparable. Dermatologists at both sites echoed concerns about referral content. There were no notable disagreements; indeed, the themes (lack of detail, common conditions being referred) were common to both.

## Discussion

This study reveals a clear disparity between foundation year doctors' self-confidence in dermatology and dermatologists' expectations and observations of referral quality. New doctors do not feel well-prepared to deal with inpatient skin problems, and dermatologists are noticing the consequences of this in the referral content they receive. The multi-site survey underscores that this is a widespread issue likely rooted in how dermatology is taught at undergraduate and early postgraduate levels.

Impact of undergraduate dermatology training

The results strongly suggest that current undergraduate medical training in dermatology is insufficient to produce doctors confident in basic dermatological assessment. Despite all UK medical schools including some dermatology in the curriculum, the exposure is often brief. Previous audits have documented that many schools fall short of BAD recommendations for dermatology teaching [2]. Our finding that 70% of foundation doctors were unaware of the BAD junior doctor handbook indicates that even readily available learning tools are not reaching students effectively, perhaps due to a lack of advertisement and integration into formal teaching. The consequence is apparent: Resident doctors struggle with fundamental tasks like describing a rash or identifying conditions like eczema flares. This is echoed by Aldridge et al., who directly measured this disconnect between training and competence, finding that medical students' diagnostic accuracy for skin lesions increased from just 11% to 33% after their dermatology attachment, with half of this improvement lost after 12 months, thus persisting into the foundation years [12].

Hussain et al. similarly found that resident doctors lacked confidence in basic dermatology, even right after medical school [4]. Our study, conducted years later, unfortunately indicates little improvement - many foundation doctors still feel inadequately prepared. Some lack of confidence is expected in new doctors unfamiliar with the specialty. However, the lack of confidence reported (e.g., two-thirds not confident managing simple skin treatments) is concerning for patient care.

The educational challenges are further complicated by institutional barriers. As Khalil and Bala demonstrated through curriculum mapping at their institution, many medical schools lack structured approaches to ensure dermatology learning outcomes are adequately covered and assessed [13]. This systematic gap in curriculum design contributes to the confidence deficits we observed.

Referral shortcomings and dermatologists' expectations

Dermatologists in our survey pinpointed specific referral shortcomings: missing history, lack of description, and no differential diagnosis, which match known issues from other studies. Cnudde et al. highlighted that 41% of inpatient referrals had no differential provided and only 29% had a correct diagnosis in the referral [6], which mirrors our specialists' concerns about "everything is a rash" with no nuance. In our results, foundation doctors admitted their uncertainty in these areas. This likely translates into incomplete referrals.

Another referral shortcoming is the management prior to referral. Ideally, referring teams should initiate basic appropriate treatment for skin conditions when possible (e.g., start emollients for eczema). Our survey found resident doctors have low confidence in using even simple treatments like emollients; correspondingly, dermatologists noted that often no treatment is started, or suboptimal treatments are used. This is corroborated by Cnudde et al., who found only 44% of referrals mentioned any treatment had been started [6].

Implications for patient care

This mismatch may impact care. First, delays in care - if referrals are missing key information, the dermatologist might need to gather that information (through phone calls or waiting until review) before making decisions, which can slow down diagnosis and treatment. Second, unnecessary referrals - conditions like uncomplicated eczema or scabies may overwhelm services, diverting specialist attention from more complex cases, simply because the resident doctor wasn't confident to treat them.

Third, patient outcomes could be impacted. A resident lacking these skills may delay recognising red flags in a genuine emergency (like severe cutaneous adverse reactions such as toxic epidermal necrolysis) or conversely, might make a referral for minor chronic conditions that might not require acute dermatologist input. This can result in delays in diagnosis and treatment for potentially life-threatening conditions. Aldridge et al. also demonstrated that inadequate dermatology training affects patient safety, as students and resident doctors fail to develop the pattern recognition skills needed for accurate diagnosis [12]. While our study didn't measure outcomes, the low confidence in identifying urgent dermatoses is worrisome.

Educational interventions and recommendations

The findings point towards several educational and system interventions.

Enhanced Undergraduate Curriculum

Medical schools should revisit their dermatology teaching to ensure it covers not only common conditions but also practical skills like describing lesions and formulating differentials. More clinical exposure, such as integrated dermatology clinics or ward rounds, could help. The literature suggests that students who had longer dermatology placements felt more prepared as FYone doctors [5]. The national survey by Chiang et al. further demonstrated that teaching by dermatology specialists and small-group learning significantly improved confidence levels [14].

Foundation Programme Training

For current resident doctors, targeted workshops or teaching sessions in dermatology could be invaluable. For instance, a short course at the start of FYone on common inpatient skin presentations, how to describe them, red flags, and how to start initial management (such as the choice of emollients, topical steroids, or need for diagnostics such as skin swabs) could increase confidence. The implementation of such programmes requires addressing systemic barriers, as outlined by Sharma et al., including securing protected educational time and appropriate resources [10].

Promotion of Resources

The low awareness of the BAD handbook suggests that information is not being disseminated. Foundation schools and postgraduate medical departments should actively promote this valuable resource, such as by distributing the PDF or link to all new FYones during induction. They could also direct them to other learning resources, such as online dermatology modules run by the British College of Dermatology.

Referral Guidelines and Templates

Hospitals could develop a standard template or checklist for inpatient dermatology referrals to ensure key information is included. For instance, an electronic referral form might prompt the doctor: "Describe lesion (morphology, distribution)", "List all medications and highlight any that have been recently started", "Differential diagnosis if known", "Urgency". The descriptive algorithm developed by Middleton and Dolan provides an excellent example of how structured tools can improve referral accuracy [9].

Mentorship and Consultation Feedback

Another approach is for dermatology teams to provide feedback on referrals as a learning loop. For example, dermatologists could send quarterly emails on common referral issues and advice on how to send a better referral. Interestingly, in a national survey of UK medical students, Chiang et al. found that those who received dermatology teaching from specialists reported higher confidence levels than those taught by generalists. Their study also demonstrated the importance of learning in a clinical setting, using evidence-based teaching approaches to address the confidence gaps in trainee doctors [14].

Several innovative approaches address these needs. Middleton and Dolan developed a descriptive algorithm for skin lesions that improved the accuracy of referral terminology from 37% to 70% when implemented as an educational intervention [9]. This kind of structured approach could be particularly valuable given our finding that foundation doctors struggle with accurate dermatological descriptions.

Technology-based solutions also show promise. Giavina-Bianchi et al. demonstrated that teledermatology systems can serve dual purposes: improving access to specialist care while providing educational opportunities for primary care physicians through structured image capture and feedback processes [15]. Such systems could be adapted for foundation doctor training, providing immediate learning opportunities during the referral process.

Comparison with other studies

Our results echo many observations from prior research in the UK and overseas. The low diagnostic agreement rate (29%) in Cnudde's Welsh referral study [6] is one quantifiable indicator of the knowledge gap that our residents qualitatively expressed. Comparable figures have been reported outside the UK. For example, an Irish audit of 703 inpatient consultations found dermatologists revised the admitting diagnosis in 55% of cases, with more than a third of referral forms offering no provisional diagnosis at all [8]. 

Unsurprisingly, confidence surveys repeatedly document the fallout - Only 20% of UK resident doctors feel able to describe a rash and eight percent to initiate basic therapy [4], while just nine percent of FYone doctors without a substantive dermatology placement consider themselves prepared for practice [5], and 90% of current foundation trainees admit they are not confident even to perform a full skin examination [16].

Taken together, these converging data show that dermatology remains under-emphasised throughout general medical training in multiple healthcare systems, not just within the UK. Our study specifically adds to the literature by directly juxtaposing resident doctor self-assessment with dermatologist expectations in the same setting. Many studies look at one side or the other (e.g., residents' confidence surveys or audits of referral letters). By capturing both, we have shown that the areas residents feel weakest in are indeed the areas dermatologists note deficiencies in practice.

Rehman et al. highlighted similar findings, specifically regarding facial skin malignancies, reporting that 90% of foundation doctors were not confident in performing full skin examinations, and 87% were not confident in investigating facial skin cancers [16]. This aligns with our broader findings on dermatological confidence and reinforces the need for more structured education.

Limitations

There are limitations to consider. The sample size of the dermatologists was small (reflecting the small number of them at each site), and their feedback was qualitative. The foundation doctor survey, while larger, is still only two hospitals; although we believe these are illustrative of many UK hospitals, a broader national survey would be useful for generalisation. The study relied on self-reported confidence rather than objective knowledge tests or observed behaviours. We also did not follow actual referral outcomes or patient results. Our conclusions about implications are inferred from the content of referrals and known literature.

As noted in other studies [5,14], there may be discrepancies between self-reported confidence and actual competence. Foundation doctors might either over-estimate or under-estimate their abilities compared with objective performance. Future work could address this by directly evaluating referral quality against objective criteria or by testing doctors' knowledge alongside confidence ratings.

## Conclusions

Newly qualified doctors in our multi-site survey reported low confidence in core dermatology skills, while dermatologists noted inpatient referrals often lack necessary detail. This mismatch risks patient care quality in the inpatient setting and highlights the need for enhanced dermatology education at undergraduate and foundation levels. Expanding curricula, targeted teaching and more accessible resources could help foundation doctors manage common skin conditions, recognise emergencies, and improve referral quality. In turn, this would allow dermatology services to function more efficiently, allowing specialists to focus on complex cases and generalists to handle routine care.

This study highlights a systemic issue requiring action from educators, training programmes, and professional bodies. Strengthening dermatology competencies in resident doctors will ultimately translate into getting it right first time, more appropriate treatments, and improved outcomes. Curriculum implementation relies on both medical school engagement and support for teaching leads through adequate resources and training.

## Appendices

Appendix A

**Table 3:** Questionnaire for resident doctors The Likert scale [11] was used by the participants to quantify their confidence.

Question	Response options
Do you feel confident in describing abnormal skin?	1-5 Likert scale (1 not at all confident, 5 extremely confident)
Are you confident in identifying red flag skin presentations?	1-5 Likert scale (1 not at all confident, 5 extremely confident)
Do you feel confident with what information to include when making an inpatient dermatology referral?	1-5 Likert scale (1 not at all confident, 5 extremely confident)
Are you confident in the different forms of emollients and when to use them?	1-5 Likert scale (1 not at all confident, 5 extremely confident)
Are you aware of the free BAD handbook for medical students and junior doctors?	Yes/No

Appendix B 

**Table 4:** Northampton resident doctors' responses to "Do you feel confident in describing abnormal skin?" The Likert scale [11] was used by the participants to quantify their confidence.

Confidence	Likert Scale	Number of responses
Not confident at all	1	6
	2	8
	3	18
	4	2
Extremely confident	5	1

Appendix C 

**Table 5:** Basildon resident doctors' responses to "Do you feel confident in describing abnormal skin?" The Likert scale [11] was used by the participants to quantify their confidence.

Confidence	Likert Scale	Number of responses
Not confident at all	1	7
	2	20
	3	21
	4	3
Extremely confident	5	1

Appendix D 

**Table 6:** Northampton resident doctors' responses to "Are you confident in identifying red flag skin presentations?" The Likert scale [11] was used by the participants to quantify their confidence.

Confidence	Likert Scale	Number of responses
Not confident at all	1	4
	2	9
	3	16
	4	5
Extremely confident	5	1

Appendix E 

**Table 7:** Basildon resident doctors' responses to "Are you confident in identifying red flag skin presentations?" The Likert scale [11] was used by the participants to quantify their confidence.

Confidence	Likert Scale	Number of responses
Not confident at all	1	4
	2	13
	3	20
	4	13
Extremely confident	5	2

Appendix F 

**Table 8:** Northampton resident doctors' responses to "Do you feel confident with what information to include when making an inpatient dermatology referral?" The Likert scale [11] was used by the participants to quantify their confidence.

Confidence	Likert Scale	Number of responses
Not confident at all	1	3
	2	13
	3	11
	4	6
Extremely confident	5	2

Appendix G 

**Table 9:** Basildon resident doctors' responses to "Do you feel confident with what information to include when making an inpatient dermatology referral?" The Likert scale [11] was used by the participants to quantify their confidence.

Confidence	Likert Scale	Number of responses
Not confident at all	1	5
	2	15
	3	9
	4	10
Extremely confident	5	2

Appendix H 

**Table 10:** Northampton resident doctors' responses to "Are you confident in different forms of emollients and when to use them?" The Likert scale [11] was used by the participants to quantify their confidence.

Confidence	Likert Scale	Number of responses
Not confident at all	1	8
	2	16
	3	10
	4	1
Extremely confident	5	0

Appendix I

**Table 11:** Basildon resident doctors' responses to "Are you confident in different forms of emollients and when to use them?" The Likert scale [11] was used by the participants to quantify their confidence.

Confidence	Likert Scale	Number of responses
Not confident at all	1	16
	2	17
	3	13
	4	4
Extremely confident	5	1

Appendix J

**Table 12:** Northampton resident doctors' responses to "Are you aware of the free BAD handbook for medical students and junior doctors?"

Response	Number of responses
Yes	11
No	24

 Appendix K

**Table 13:** Basildon resident doctors' responses to "Are you aware of the free BAD handbook for medical students and junior doctors?"

Response	Number of responses
Yes	15
No	37

Appendix L 

**Table 14:** Questionnaire for dermatologists

Question	Response option
What information would you like to have when you review an inpatient referral?	Open text
What information do you commonly find to be lacking within inpatient referrals?	Open text
Are there any recurring inappropriate inpatient referrals which you believe could be resolved with better education? E.g specific conditions. Please specify.	Open text

Appendix M

**Table 15:** Northampton dermatologists’ responses to the questionnaire

Questions	Responses
What information would you like to have when you review an inpatient referral?	-Detailed history about the skin condition especially onset, duration, distribution, stable or progressive, symptoms like itching, burning, any associated systemic symptoms, past history of skin condition. Treatment used Photography taking from a distance showing the distribution of rash as well close up photos. -Reason for referral, brief hx and examination findings -Details of the presenting complaint, site distribution, duration, characteristics and any associated symptoms. Any past history or family history. Medical and drug history
What information do you commonly find to be lacking within inpatient referrals?	-Limited information about progression and distribution of rash -Differential diagnosis -All the usual history needed.
Are there any recurring inappropriate inpatient referrals which you believe could be resolved with better education?	-Chronic skin condition that are not making patient unwell or preventing discharge. These can be managed in primary care by GP who can refer to Dermatology through proper pathway. -Yes to some extent -Bilateral cellulitis

Appendix N 

**Table 16:** Basildon dermatologists’ responses to the questionnaire

Questions	Responses
What information would you like to have when you review an inpatient referral?	-Patient medical history, drug history, reason for admission -Details of the presenting complaint including onset, associated symptoms, medications used so far. Any systemic complaints -Accurate description of the problem, time scale and patient details including reason for admission. -Urgency (does this really require an inpatient review or can this be sent as an outpatient referral!), differentials, previous derm treatment if known to derm, photos if possible and to attempt describing the rash -Comorbidites.
What information do you commonly find to be lacking within inpatient referrals?	-Previous medical history details and detail history of drugs patients taking -Details of the presenting complaint -Accurate description! Everything is a rash!! -The above -Medications
Are there any recurring inappropriate inpatient referrals which you believe could be resolved with better education?	-Rashes, type of rash, onset of rash and details, drug history, associated medical conditions -None -Drug eruptions, frequent cause of referral for no particular reason -Psoriasis, eczema, scabies, leg ulcers -Leg ulcers should be referred only after leg ulcer team management and TVN input.
